# A case report of hepatic angiomyolipoma, inflammatory subtype. Clinico-pathological characterization: a diagnostic challenge

**DOI:** 10.1186/s13000-023-01343-7

**Published:** 2023-04-28

**Authors:** Francisco Javier Velasco-Albendea, María Jesús Gil-Belmonte, Beatriz Estébanez-Ferrero, Orlando Fuentes-Porcel, Bruno José Nievas-Soriano

**Affiliations:** 1 Department of Pathology, Torrecardenas Hospital, Almería, 04009 Spain; 2 Department of General Surgery and Digestive Diseases. Torrecardenas Hospital, 04009 Almería, Spain; 3 Nursing, Physiotherapy, and Medicine Department, University of Almería, 04120 Almería, Spain

**Keywords:** Angiomyolipoma, Liver, Immunoglobulin G4-Related disease, Inflammatory

## Abstract

Angiomyolipoma is a benign mesenchymal neoplasm of a wide histological heterogeneity belonging to the PEComa “family.” The liver, after the kidney, is their second most frequent location. However, inflammatory hepatic AMLs constitute a rare entity, with only fourteen documented cases until 2020. These neoplasms can overlap morphological features of IgG4-related diseases, being of great diagnostic relevance to demonstrating myomelanocytic-lineage differentiation of the neoplastic cells. Case presentation: we present a new case of an inflammatory hepatic AML resembling an IgG4-related disease in a 35-year-old woman with a subcapsular 5 cm mass confined to segment VII of the right hepatic lobe. Although having reduced its size along the tumor’s natural evolution, complete tumor resection was decided due to its hypermetabolic behavior (max. SUV = 12,6) assessed by PET-CT scan. Finally, the patient underwent a right hepatectomy due to spontaneous rupture and bleeding of the lesion during the intervention. All the diagnostic and therapeutic procedures occurred in the last months of the COVID-19 pandemic. Conclusions: This review aims to describe inflammatory hepatic AML histological and immunohistochemical features. We further sought to establish a clinicopathological contextualization of this tumoral subtype.

## Introduction

First described in 1976 [1], hepatic AML angiomyolipoma (AML) is a solid mesenchymal tumor included in the group of PEComas (tumors derived from perivascular-epithelioid cells) of a usually benign nature [1–11]. Given its asymptomatic behavior, most of them occur as incidental findings. Occasionally, they can be associated with weight loss, abdominal pain, swelling, or spontaneous rupture. AML rarely appears as a palpable mass. The classically described relationship with tuberous sclerosis is only observed in 5–15% of the cases, contrary to renal AML (up to 50% of the cases). Bleeding due to spontaneous rupture is even more unusual than in kidneys [3]. As the name reads, AML hosts a morphological substrate of myoid, mature adipose tissue, and thick-wall poorly formed vessels in different proportions, giving this entity a highly variable histological spectrum. Morphological patterns described in the literature include the following: lipomatous, myomatous, angiomatous, trabecular, pelioid, inflammatory, and mixed patterns [4, 6–11]. The inflammatory subtype was first published in 2004 [12]. Since then, only fourteen cases have been reported and are available in Medline [4]. 2019 World Health Organization (WHO) Classification currently regards inflammatory hepatic AML as a distinct subtype [2].

Inflammatory hepatic AML, according to the existing literature, most frequently affects women with an average age of 46,5 years. Commonly found in the left hepatic lobe, inflammatory AML’s average size is 6,65 cm. Abdominal pain and discomfort are the typical clinical manifestations, and no association with tuberous sclerosis has been described [3, 4]. Imaging techniques reveal a well-defined and hypervascular solid mass. Differential diagnosis includes other primary benign or malignant lesions and metastases, making it a diagnostic challenge [4–9, 11].

Although the exceptionally aggressive course of this disease has been reported, there are no examples of malignant inflammatory hepatic AML [3–5]. Surgical treatment of hepatic AML is often curative, particularly in the inflammatory subtype, as twelve reported cases were disease-free after surgery. Of the two remaining cases, one showed no recurrence with arterial embolization, and the other, diagnosed by biopsy, had no follow-up data [1–11, 13, 14].

## Case description

**Fig. 1:**
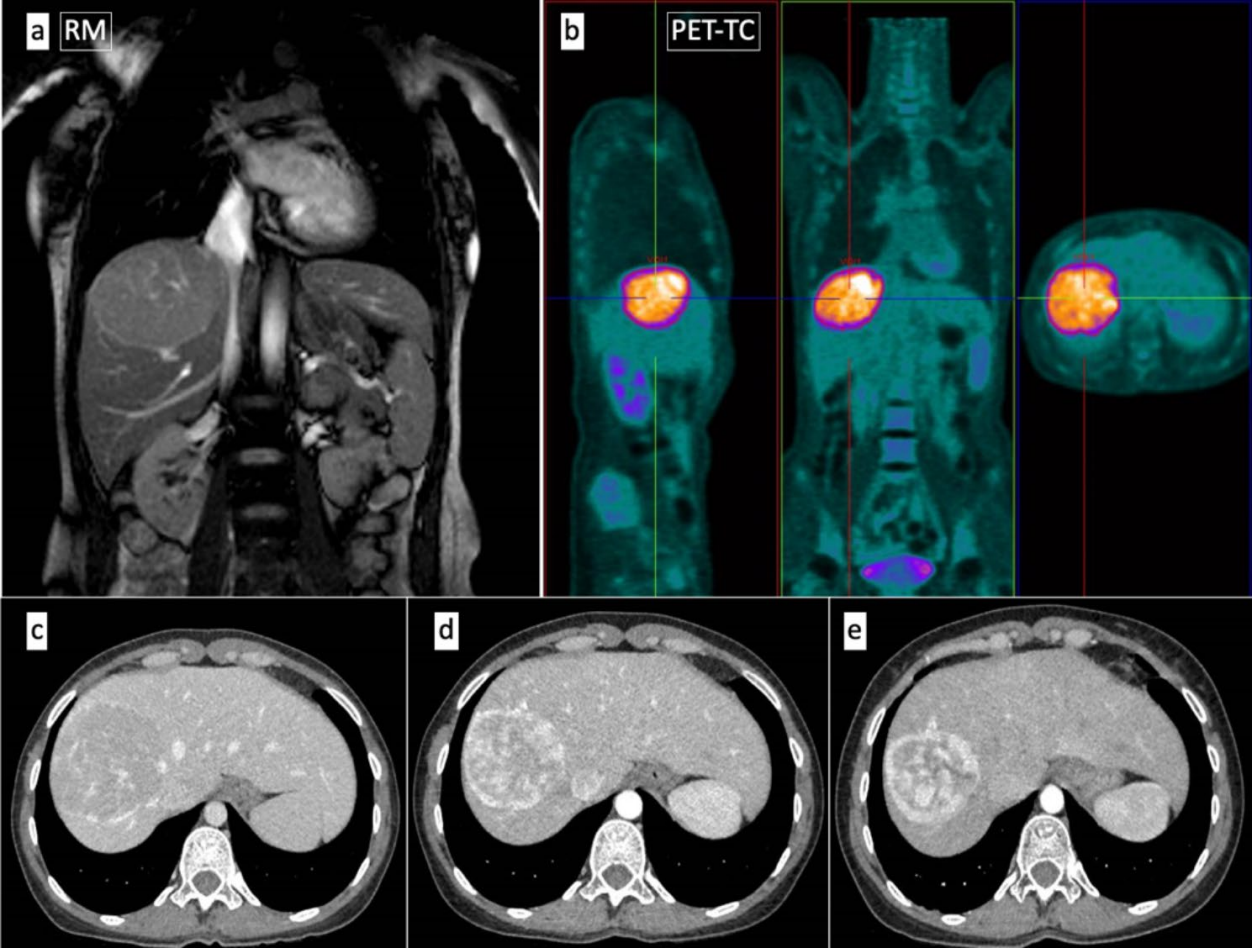
MRI image of a solid mass involving segment VII of the right hepatic lobe (**a**). Hypermetabolic, hypodense, and homogeneous hepatic lesion (SUV max = 12,6) of demarcated boundaries (**b**). Comparative study of contrast-enhanced CT-scan every two months with a gradual reduction in size over time (**c, d**, and **e**)

**Fig. 2:**
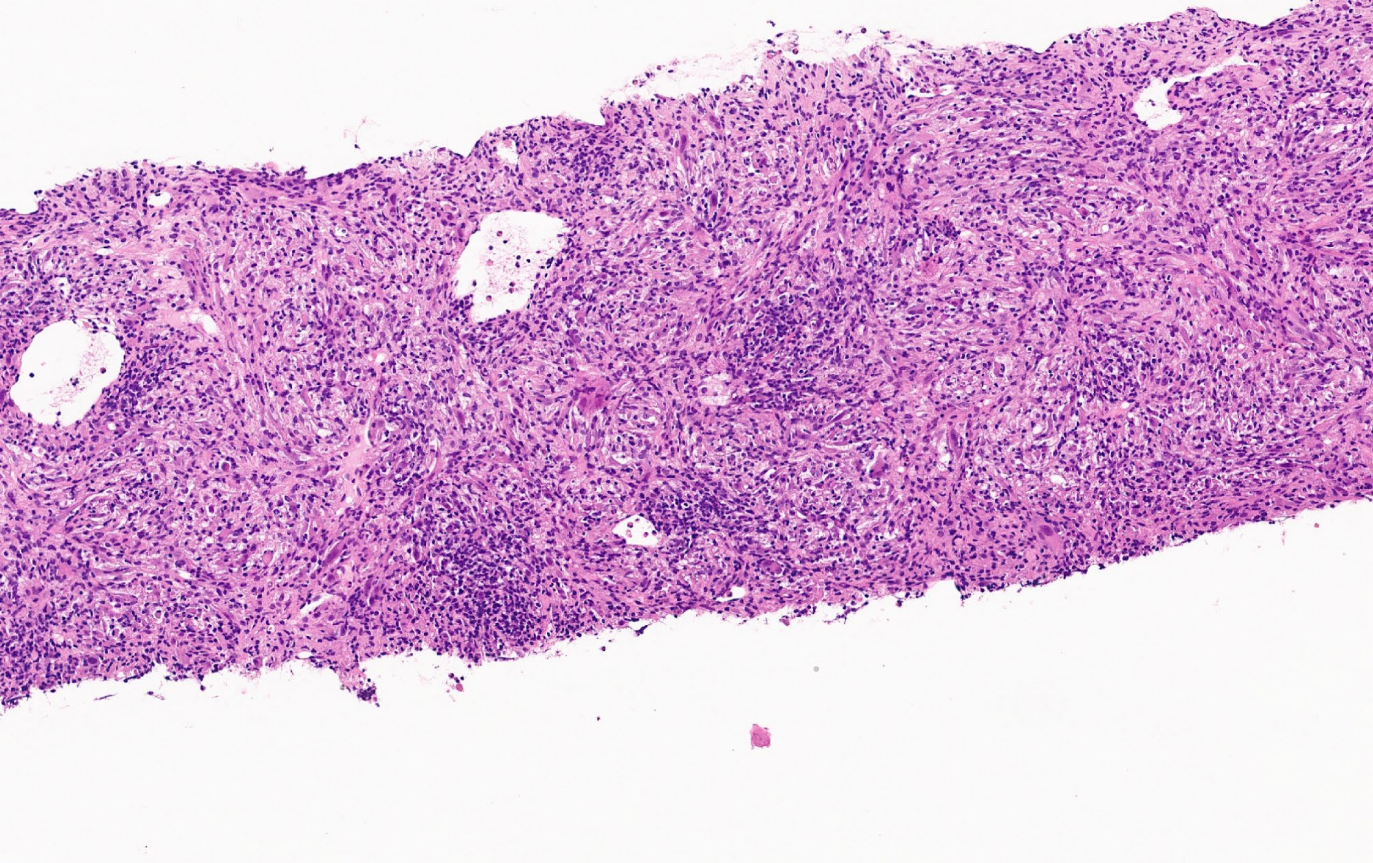
Core needle biopsy (CNB). A mesenchymal spindled cell proliferation confirming crossed bundles associated with a lymphoplasmacytic infiltrate

The case of a 35-year-old woman with a manifest lack of appetite, weight loss, asthenia, general malaise, and digestive discomfort is presented. The blood test revealed normocytic and normochromic anemia, thrombocytosis, and slight hypereosinophilia. The serological analysis showed positive IgM for Rickettsia coronii and indeterminate IgM levels for Mycoplasma pneumoniae. Treatment with doxycycline, 100 mg twice per day, was given. The evolution was a clinical improvement but without a complete resolution of the symptomatology. Ultrasonographical studies of the abdominal cavity detected a hypoechogenic lesion resembling a hepatic abscess. It affected the posterior aspect of the right hepatic lobe, measuring 8,4 cm x 6 cm. Contrast-enhanced CT scan describes this lesion as a solid image of 8 cm with peripheral hypervascularization with the radiological orientation of a hepatic adenoma. Finally, contrast-enhanced dynamic MRI defined it as a well-demarcated lesion of 7,8 cm (AP) x 9,3 cm (RL) x 6,7 cm (CC) with portal vessel deviation, T1-hypointense signal and intermediate signal in T2 compared to the normal hepatic parenchyma. A perfusion decrease was also observed, giving the diagnostic impression of a hepatic adenoma, so clinical follow-up was strongly recommended. However, the PET-CT scan evidenced a hypermetabolic behavior of SUVmax = 12,6, establishing the need to exclude a malignant lesion (Fig. 1a and b. Thus, a core needle biopsy (CNB) was performed at another clinical institution. The first diagnostic approach was an inflammatory pseudotumor / IgG4-related disease (IgG4-RD) versus an inflammatory myofibroblastic tumor. The latter option was excluded due to its negative staining for ALK. A mesenchymal spindled cell proliferation of crossed bundles associated with a prominent lymphoplasmacytic infiltrate was already described at the CNB (Fig. 2). IgG-positive plasma cells accounting for more than 10 per high-power field favored the possibility of an IgG4-RD. A more exhaustive serological analysis was conducted. It evidenced a serum IgG4 of 245,7 mg /dL (normal: 3,92–86,4 mg/dL) with normal IgG and autoimmunity studies.

**Fig. 3:**
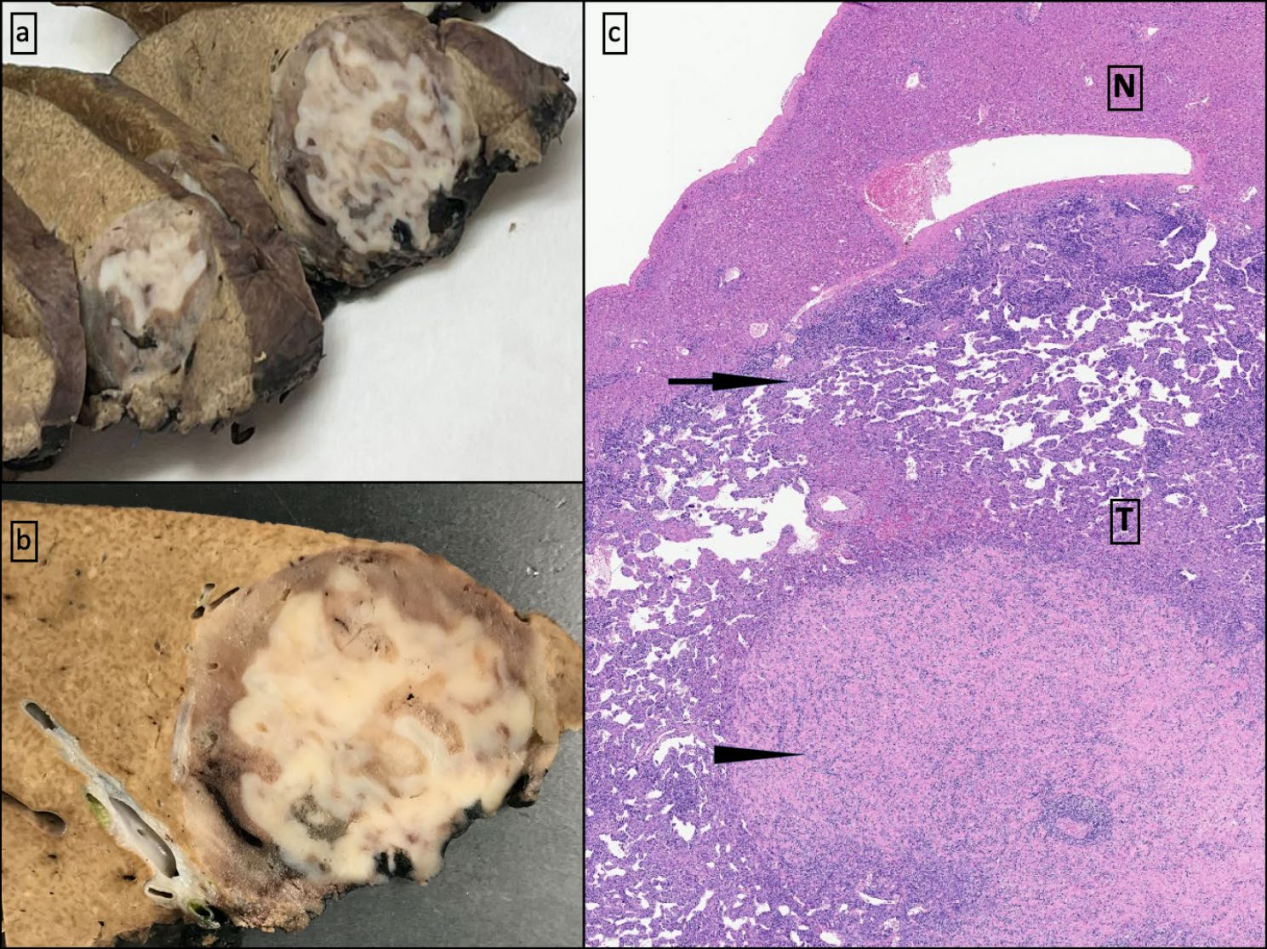
Hepatic parenchyma sections with a rounded and solid tumoral lesion of striped appearance (**a** and **b**). Panoramic macroscopic-microscopic view of normal tissue of the liver (N) and tumor (T). Two different components can be identified: the hypercellular and vascularized zone (arrow) corresponding to macroscopic brownish bands and the main sclerosing hypocellular region (arrowhead) **(c)**

Close clinical follow-up was initially decided. A spontaneous decrease in the tumor size was demonstrated by a CT scan two months later with a 7,1 cm variation in the long axis and homogeneous contrast enhancement at the arterial phase. Two months later, the lesion kept reducing, reaching a size of 6,5 cm (Fig. 1c, d, and e). Given its persistence and its uncertain metabolic behavior, resection was performed. A spontaneous capsule rupture occurred during the procedure, leading to bleeding and subsequent hemodynamic instability. As a result, conservative surgery was discarded, opting for a right-lobe hepatectomy of 17 × 12 × 5 cm weighing 428 g and containing the tumor. The macroscopic study revealed a white-colored solid mass composed of intermingled bands measuring 5 cm x 4,5 cm with well-defined contours and elastic consistency, giving it a striped appearance (Fig. 3a,b and c).

**Fig. 4:**
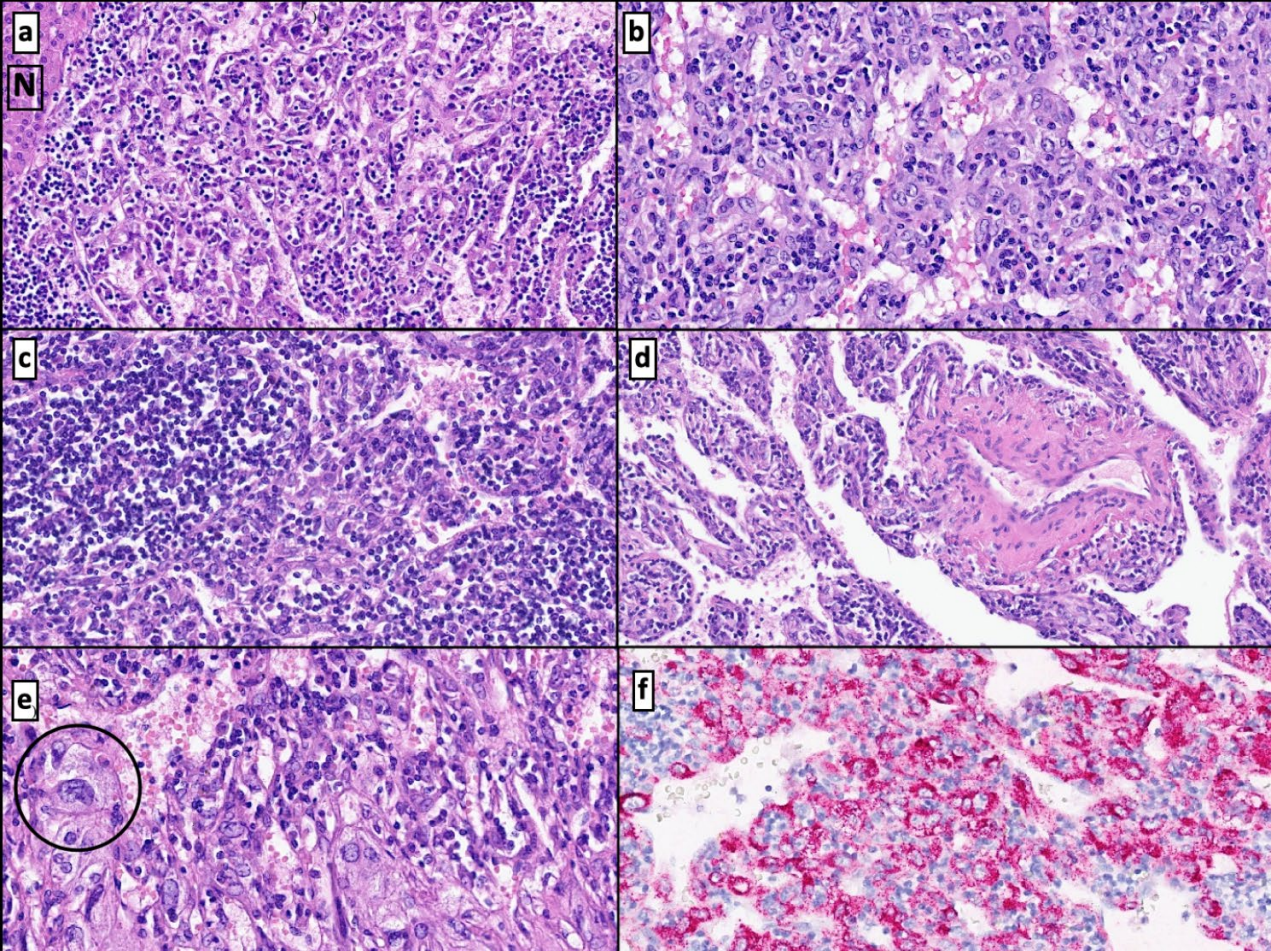
Solid neoplasm with vascular channels encircled by normal liver parenchyma (N) of well-defined borders **(a)**. The lesion shows pseudovascular patterns with abundant lymphocytes, perivascular eosinophils, and epithelioid cells **(b)**. Tumor-associated lymphohistiocytic infiltrate **(c)**. Thick-wall vessels and dilated vascular channels with thin septae hosting tumoral cells **(d)**. Detail of neoplastic “PEComatous” epithelioid cells of large nuclei neighboring vascular areas (circle) **(e)**. Positive HMB-45 immunoexpression of tumor cellularity **(f)**

**Fig. 5:**
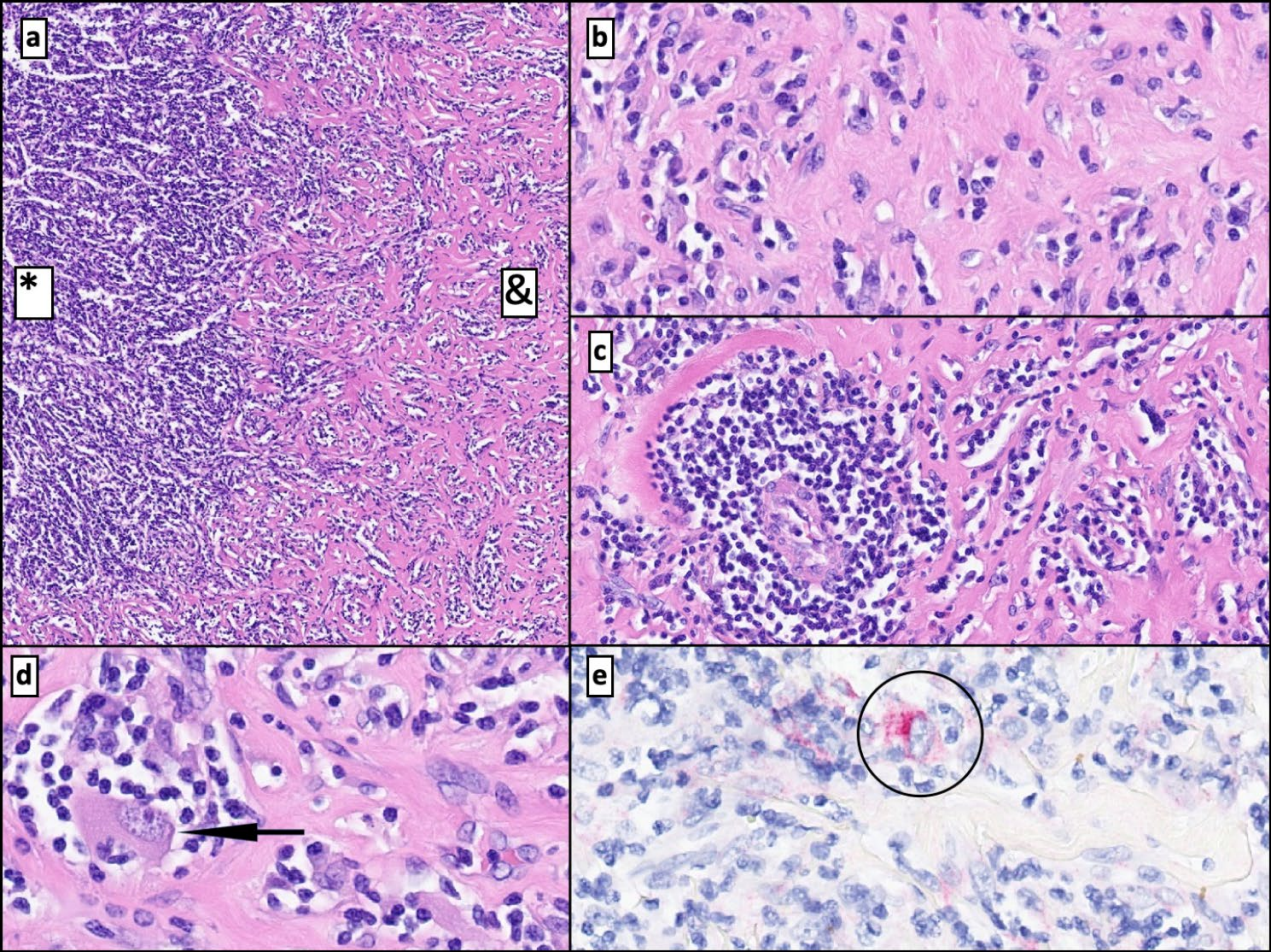
Interphase zone with an inflammation-rich pseudovascular area (*) and a fibrous and sclerosing component (&) **(a)**. Fibrous neoplastic tissue with sparse epithelioid cells within the lymphoplasmacytic infiltrate **(b)**. Vessel surrounded by lymphoplasmacytic cells in the fibrosclerotic region **(c)**. Detail of a neoplastic cell: eosinophilic cytoplasm with distinct nucleoli next to lymphocytes and plasma cells (arrow) **(d)**. Cytoplasmic positivity for MelanA in a single cell in the fibrosclerotic region (circle) **(e)**

**Fig. 6:**
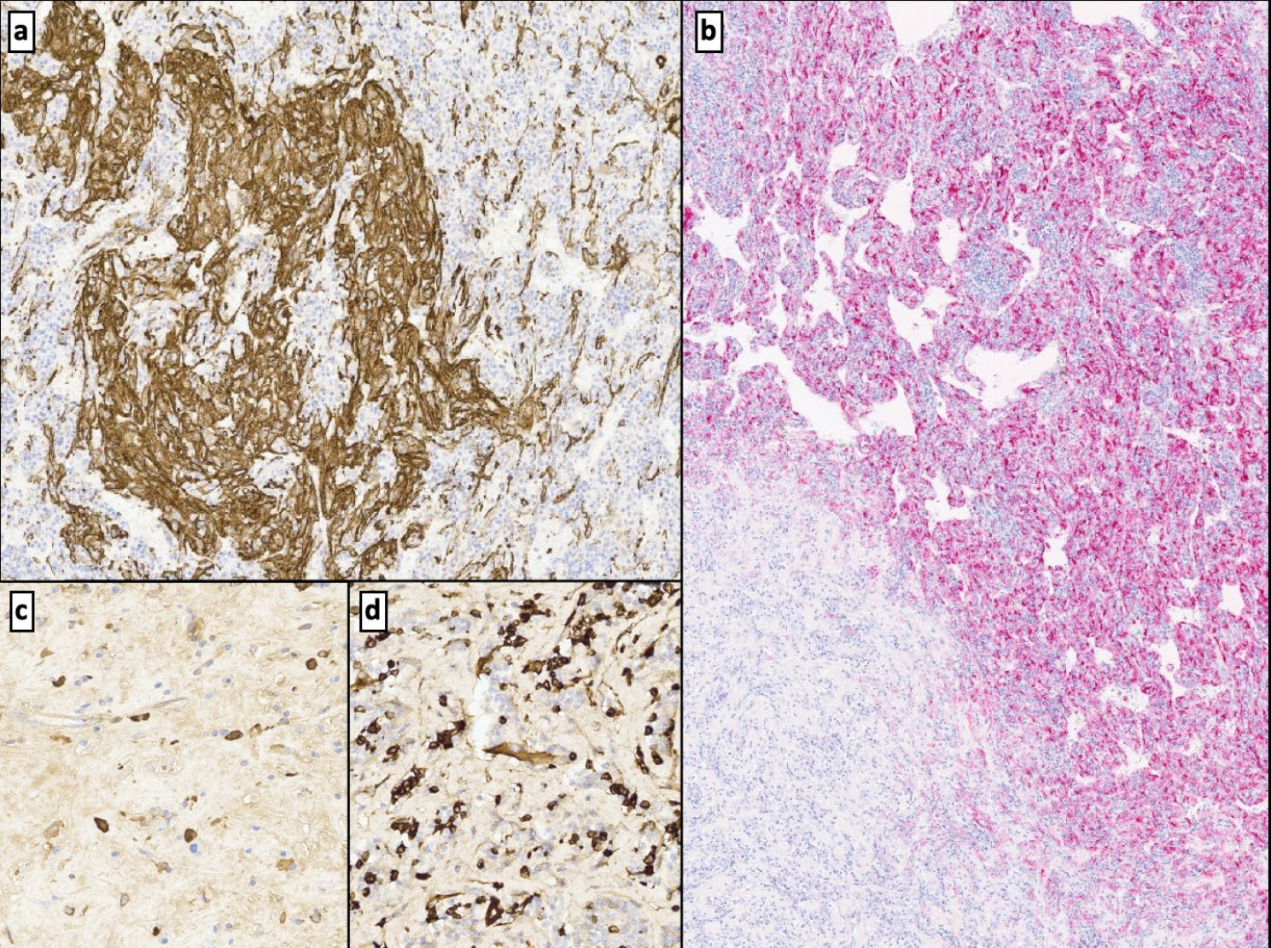
Positive immunostaining for SMA in the epithelioid tumor cells **(a)**. HMB-45 immunoexpression in the pseudovascular areas of the neoplasm (diffuse positivity) and fibrous and sclerosing regions (single cell positivity) **(b)**. Plasma cells expressing IgG4 **(c)** and IgG **(d)**

Microscopic observation showed a mesenchymal neoplasm of subcapsular and intraparenchymal location. This lesion had demarcated boundaries and was separated from normal hepatic tissue by a fibrous pseudocapsule with two periodically alternating zones explaining its macroscopic striped appearance. One of these alternating zones (Fig. 4a, b, c, d and e) had vascular channels. It conferred a pseudovascular/sinusoidal-cavernous impression and a prominent lymphocyte infiltrate (type B and, mostly, type T cells) together with mature plasma cells, histiocytes, and thin fibrous septa. In addition, perivascular eosinophilic, epithelioid, spindled cells of a myo-histiocytoid habit were seen. These cells exhibited large nuclei, binucleations, and distinct nucleoli. The immunohistochemical analysis demonstrated cytoplasmatic positivity for vimentin. Specific smooth muscle actin (SMA) and a heterogeneous expression of CD68 were also shown. An intense immunoexpression of melanocytic markers like MelanA and HMB-45 (Figs. 4f and 5e, and 6a and b) was present.

Immunoexpression was negative for HepPar-1, alpha-fetoprotein, CD56, NeuN, synaptophysin, chromogranin, pan-cytokeratin, CD117, ALK, S-100, and SOX10. Other evaluated techniques, such as Glypican 3, WT-1, CD15, CD30, CK8/18, β-hCG, and inhibin-alpha, were also negative (Table 1).

**Table 1:** Summary of immunohistochemical studies

Panel of Immunohistochemical Stains in tumor cells	
Immunohistochemical Stain	Result
Vimentin	Positive
Smooth muscle actin (SMA)	Positive
CD68	Heterogeneous expression
Melan A	Positive
HMB-45	Positive
HepPar-1	Negative
Alpha-fetoprotein	Negative
CD56	Negative
NeuN	Negative
Synaptophysin	Negative
Chromogranin	Negative
Pan-cytokeratin (AE1-AE3)	Negative
CD117	Negative
ALK	Negative
S-100	Negative
SOX-10	Negative
Glypican	Negative
WT-1	Negative
CD15	Negative
CD30	Negative
CK8-18	Negative
B-hCG	Negative
Inhibin-Alpha	Negative

The second distinguishable tumoral area corresponds to a fibroesclerotic tissue, exhibiting a swirling pattern and hyalinized stroma of lessened cellularity, hosting a minor inflammatory infiltrate consisting of histiocytes, sparse eosinophils, frequent mature plasma cells (CD138+) and the same vaguely “Reed-Sternbergoid” cells with a mixed myomelanocytic phenotype. In this area, cells were alone or grouped in small aggregates (Fig. 5a, b, c, and d). Plasma cells had no lambda nor kappa (polytype) chain restriction and showed immunoexpression for IgG and IgG4 (IgG4 + plasma cell /high-power field (HPF): 8–12 /HPF and IgG4/IgG ratio: < 40%) (Fig. 6c and d).

Additionally, thickened walls were spotted at the periphery, with no evidence of fatty tissue in the tumor sample. Prominent lymphoplasmacytic infiltrate surrounding vessels was evidenced. Some signs of luminal obliteration at the fibrosclerotic areas were also seen (Fig. 5c). Sporadic dystrophic microcalcifications were also present. No granulomas or extramedullary hematopoietic foci were identified. No remarkable changes were seen in the normal liver.

Low mitotic activity was observed (0–1 mitosis per 50 HPF). The inflammatory infiltrate globally accounted for more than 50% of the lesion. No necrosis nor lymphovascular invasion phenomena occurred. Even though tumor borders were expansive, resection margins were free of neoplasm.

## Discussion

AML is a rare mesenchymal neoplasm of a usually benign biological behavior composed of a heterogeneous mix of blood vessels, smooth muscle /myoid cells (epithelioid and fusiform), and mature adipose tissue. It commonly develops in the kidney, followed by the liver in frequency [3–11]. Radiology image-based diagnosis can be complex, especially when there is a low or absent adipose tissue proportion, as is our case [3, 5, 7, 13, 14]. Hepatic AML, the inflammatory subtype, has the peculiarity to show a prominent inflammatory infiltrate microscopically. It comprises lymphocytes, plasma cells, and histiocytes and holds the diagnostic key. This inflammatory cellularity represents more than 50% of the tumor surface, “hiding” typical and distinctive AML features [1–11, 13].

Inflammatory AML of the liver, described by Kojima M. in 2004 [12], is a pathological entity with remarkable characteristics that need to be identified to consider this diagnostic possibility, as is the current case we propose. Comparing it to the other fourteen hepatic inflammatory AMLs reported until now, similar features help categorize this subtype: the least frequent of hepatic AML [3–10, 12–14]. If our case is added to the previously published ones, grouped by Jia-Xi Mao [4], 12 cases in female patients would be confronted with three occurring in males. Symptomatology is consistent with the customary: abdominal discomfort and anorexia. Five centimeters tumor size is within the range proposed (1,3 cm to 11,5 cm with an average value of 6,65 cm). With this new contribution, the overall laterality distribution remains more balanced, as a new case for the right hepatic lobe adds to the list resulting in 8 neoplasms affecting the left hepatic lobe versus 7 compromising the right hepatic lobe. Tumor boundaries are demarcated in 13 of 14 published cases, as in ours. It shares with the other 14 cases the existence of an inflammatory infiltrate associated with the neoplasm, which involves more than 50% of the tumor section. Regarding the constitutive elements of AML, our case does not show adipocytes, as proven in 3 out of 14 AMLs [5, 13, 14]. The absence of scattered fatty vacuoles, previously described in this type of tumor, implies a greater diagnostic difficulty. One of those three cases coincided with an elevation in the IgG4:IgG ratio, but it lacked prominent reticular-storiform fibrosis, and serum IgG4 levels were normal [13].

No relation with tuberous sclerosis is reported in all cases published to date. There is no evidence of disease persistence after six months post-treatment, agreeing with 12 case descriptions that refer to prognosis and that include clinical follow-up data (ranging from 2 to 108 months)[4].

The currently depicted case displays a combination of diagnostic morphological features of hepatic inflammatory AML and histological characteristics in common with IgG4-RD, agreeing with the case described by Agaimy and Märkl [6]. The tumor shows two histologically different and periodically alternating areas. They showed greater dotation of myomelanocytic cells and fibrous tissue, in both cases surrounded by a variable lymphoplasmacytic infiltrate, significantly where there is diminished fibrosis. The fibrosis-rich region is the one that resembles the most IgG4-RD histology. Additionally, there is some controversy regarding our case, which shows high serum IgG4 levels (245,7 mg/dL) (previously unreported data) [6].

IgG4-RD constitutes a fibroinflammatory systemic process with a substantial plasma cell infiltration affecting different organs (pancreas, kidneys, biliary tree, liver, salivary glands, orbit, aortic artery, or lungs). It is associated with fibrosclerosis and obliterative phlebitis. Elevated IgG4 serum levels are found in up to 50% of the cases [15–21]. Liver IgG4-RD develops as an active chronic hepatitis or a pseudoinflammatory tumor, creating the image of a mass suspicious of malignancy. The diagnosis of IgG4-RD relies upon the combination of clinical, radiological, and histopathological findings [15, 17–21]. According to the American College of Rheumatology/ European League Against Rheumatism (ACR/EULAR, 2019) [15, 17, 20], strict criteria must be sequentially met to prove or exclude the diagnosis of IgG4-RD based on a pathological-assessed score index [15, 17, 20, 21]. The cut-off point in the number of plasma cells (number of IgG4 + plasma cells per HPF) is different for each organ involved (> 50 IgG4 /HPF for the liver) [5, 6, 17, 18, 21]. The consensual agreement is reached for two requisites: > 10 IgG4 plasma cells /HPF with an IgG4/IgG ratio > 40% and serum levels of IgG4 > 135 mg /dl [16]. Our case also displays fibrosclerosis and obliterative phlebitis features, and serum IgG4 reaches pathological levels. The number of IgG4-positive plasma cells is 8–12/HPF. However, the IgG4/IgG ratio is below 40%. Considering all the inclusion and exclusion criteria enlisted by the ACR/EULAR 2019 the diagnosis of an IgG4-RD [15, 17, 19–21], our case does not reach the required score.

A histological feature of interest for is the proportion of lymphoplasmacytic inflammation, more than 50% of the tumor [1–10, 13, 14]. This aspect difficulted to distinguish our lesion from an IgG4-RD, a simple epiphenomenon, a concomitant paraneoplastic pathology, or an exacerbated immune response. The latter is constated in some cases of hepatic inflammatory AML. The only case in which the IgG4-RD relationship is suggested poses similar questions, highlighting the possibility that IgG4 could be involved in the development of the inflammatory infiltrate and stromal and vascular changes of AML [6].

We believe the similarity, possible overlap, or concomitance of inflammatory AML with IgG4-RD must be considered. It would be appropriate to perform a thorough immunohistochemical evaluation of myomelanocytic markers in liver lesions cataloged as IgG4-RD to rule out the presence of AML and serum IgG4 determinations.

In a purely speculative sense, a diagnostic dilemma arises from the striking variation in tumor size in a few months on radiological follow-up controls (from 9,3 to 5 cm). The initial doxycycline treatment with an immunoregulatory effect without corticosteroids or other immunosuppressors (despite a prior diagnostic approach of IgG4-RD by CNB) does not fully explain the size reduction. This gives added value to the uneven distribution of the lymphoplasmacytic inflammatory component of AML that we present together with the alternating pattern of sclerotic fibrosis with areas of higher cell density and with pseudovascular/sinusoidal pattern. It is fair to believe that inflammatory AML of the liver may behave as hypermetabolic (elevated SUV by PET-CT scan) and dynamic lesion depending on the variable tumor-related inflammatory response. The heterogeneous histology and macroscopic appearance of the neoplasm may respond to the process of volume variation. This dimensional heterogeneity has not been observed by the other reports in the literature from the radiologist’s point of view [1–11, 13]. Nevertheless, the small number of cases reported to date does not allow us to affirm that these tumors may undergo regressive or even progressive changes associated with the intensity of the inflammatory response of AML, nor do they have a clear association with IgG4-RD.

In our case, remission was confirmed six months after the surgical procedure. Significantly, serum IgG4 levels reached normal parameters (78.8 mg / dL) compared to the first values (245.7 mg/dL). This data would support the hypothesis that those cases of inflammatory AML with histology similar to IgG4-RD may be associated with high levels of IgG4. Although having occurred during the COVID-19 pandemic, no delays in diagnosis or treatment were observed. Additionally, no SARS-COV-2 infection was detected during the whole process by RT-PCR.

Getting familiar with this entity is relevant for an optimal differential diagnosis in hepatic tumors coursing with inflammation-rich histology, such as the inflammatory myofibroblastic tumor, the dendritic follicular cell tumor, and IgG4-RD. Well-vascularized tumors like hemangiomas should also be considered. Also, neoplasms of epithelioid-fusiform morphology, including hepatic adenoma, focal nodal hyperplasia, hepatocellular carcinoma, gastrointestinal stromal tumors, and metastases (particularly those derived from renal cell carcinomas) [4–9]. The demonstration of a cellular PEComatous component with myomelanocytic differentiation in these cases is diagnostic itself [1–10, 13, 14].

To conclude, pathologists should identify this rare subtype of inflammatory AML of the liver (with only 15 cases described to date, including ours), in which the percentage of inflammatory infiltrate is above 50%. Adipose cellular elements are sometimes absent, and the presence of vessels can be misinterpreted as non-neoplastic, which causes AML to go unnoticed. As a result, performing a targeted immunohistochemical analysis should include myomelanocytic differentiation markers to typify the neoplastic cell. Its similarity, possible overlap, or concomitance with IgG4-RD must be taken into account, being necessary to gather more documented cases to define a significant association between both entities. Finally, in our case, the variation of tumor size could be justified by a tumoral inflammatory response of variable course.

## Data Availability

The article includes all the data.

## Abbreviations

AML: Angiomyolipoma.
CNB: Core needle biopsy.
IgG4-RD: IgG4-related disease.
WHO: World Health Organization.
HPF: High power field.
ACR/EULAR: American College of Rheumatology/ European League Against Rheumatism
